# First Description of Novel End-Organ Effects by Speed Modulation Using the Aortix™ Device

**DOI:** 10.1155/2024/7430212

**Published:** 2024-04-24

**Authors:** Ajay Bhandari, Alden Dunham, Emmanuel Bassily, Bibhu D. Mohanty, Robby Wu

**Affiliations:** University of South Florida, Tampa, FL, USA

## Abstract

Aortix™ is a novel percutaneous mechanical circulatory support device designed to facilitate diuresis in patients with cardiorenal syndrome. We describe for the first time the development of end-organ hypoperfusion from excess blood acceleration at the nominal setting and demonstrate through temporal-perfusion marker curves, the potential for speed modulation to optimize results. This will inform future device development and investigation of patient-specific device titration.

## 1. Introduction

The Aortix™ device is a novel percutaneous mechanical circulatory support device currently being tested for patients with cardiorenal syndrome. We will highlight the mechanism and use of this device as well as the hemodynamics of speed modulation in the renal arteries to facilitate diuresis. We uniquely describe the novel finding that the nominal speed may not be ideal for all patients and that device speed modulation may permit patient-specific therapy optimization.

## 2. Case Presentation

**Figure 1:**
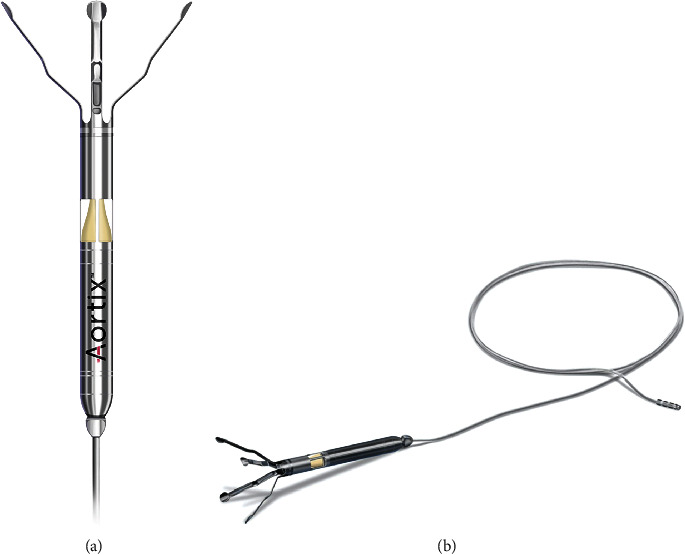
Design of the Aortix™ (manufacturer Procyrion) novel percutaneous mechanical circulatory support device. (a) Aortix™ device demonstrating its 18F axial flow structure. (b) Aortix™ device demonstrating its 6F transfemoral artery power lead and atraumatic nitinol struts for dynamic stabilization in the vessel (permission granted from Procyrion for use of copyright images). F = French.

A 52-year-old woman with nonischemic cardiomyopathy and a left ventricular ejection fraction of 10-15% presented with class 3 exertional dyspnea and anasarca. Pertinent laboratory studies showed a brain naturetic peptide level of 2,954 pg/mL, sodium level of 130 mEq/L, and serum creatinine of 1.4 mg/dL (baseline). Transthoracic echocardiography (TTE) demonstrated unchanged left ventricular dysfunction and a dilated right ventricle with moderate to severe reduction in systolic function. Initial management of acute heart failure included continuous furosemide infusion. Invasive hemodynamics measured by a pulmonary artery catheter were significantly elevated (Table 1, pre-device implantation). Despite aggressive diuretic titration, urine output remained insufficient to achieve optimal diuresis: by hospital day eight, she was net negative 10.6 L in urine output, with a mean right atrial pressure of 21 mmHg. Given a rising serum creatinine, cardiorenal syndrome was suspected. This prompted consideration of a novel percutaneous mechanical circulatory support (pMCS) device targeting management of cardiorenal syndrome—the Aortix™ pump (Figure 1). The device is positioned in the descending aorta at the level of the distal thoracic spine, projecting blood forward to provide increased perfusion between the mesentery and the renal arteries.

The device was successfully implanted through right femoral artery access, by standard implant technique. The device was turned on at the established speed of 25,000 rotations per minute (rpm); no flow or suction alarms were noted. About nine to twelve hours post-device implantation, the patient had complaints of nausea and became acutely oliguric. She developed acute kidney and liver injury with associated lactic acidosis of 3.5 mmol/L (Table 1). Due to such an acute metabolic change soon after device implantation, there was high suspicion for negative device-related effects instead of progression of persistent low output in setting of chronic nonischemic cardiomyopathy.

### 2.1. Past Medical History

1. Dilated nonischemic cardiomyopathy
2. Implantation of dual chamber cardiac defibrillator
3. Insulin-dependent diabetes mellitus, type II
4. Prior rectal cancer requiring colostomy reversal and chemotherapy
5. Obstructive sleep apnea
6. Polycystic ovarian syndrome
7. Hidradenitis suppurativa

### 2.2. Clinical/Diagnostic Hypotheses

1. Patient-specific excess rotational speed of the Aortix™ device causing inadvertent intra-abdominal organ hypoperfusion
2. Obstruction of intra-abdominal vasculature secondary to the Aortix™ device
3. Acute cardiogenic shock secondary to chronic low output state and cardiorenal syndrome
4. Septic shock due to infection from device implant
5. Septic shock due to translocation from hidradenitis blisters

### 2.3. Investigations

Repeat TTE was unchanged from prior study. Noncontrasted computed tomography (CT) scan of the abdomen and pelvis demonstrated appropriate Aortix™ device positioning, with tip termination at the T9-T10 level. Anasarca was noted. Renal ultrasound showed diffusely low flow velocities on the right side; the left side was poorly visualized. The renal artery to aortic flow ratio was unattainable due to high-grade turbulence in aortic flow, confounding waveform analysis. Liver ultrasound demonstrated severely depressed velocities in the common hepatic artery and signs of both intra- and extrahepatic congestion.

### 2.4. Management

The loop diuretic infusion was stopped, and intravenous fluid boluses were administered. The serum creatine rose to a peak of 3.2 mg/dL and lactic acid peaked at 5 mmol/L. Vasopressors were required to maintain mean arterial pressure. Blood cultures were drawn, and milrinone infusion and empiric antibiotics were started for shock of unclear etiology.

The device was appropriately positioned by CT, and there were no abnormal device alarms. Infectious work-up was unremarkable. There was suspicion for device-related excess rotational speed, causing intra-abdominal organ hypoperfusion. The device speed was therefore slowly decreased, ultimately to 21,000 rpm. The patient's urine output subsequently increased significantly. The milrinone infusion was discontinued that afternoon, and she produced 5 L urine output by that evening. RAP had improved to 8 mmHg with PAWP of 20 mmHg. 24 hours later, suction flow alarms were delivered, and the speed was increased back up to 25,000 rpm. On day four of device implantation, she had significant clinical, metabolic, and hemodynamic improvement. The device was removed, and loop diuretics were resumed with bolus dosing. Hemodynamic assessment had also significantly improved (Table 1, post-device removal).

### 2.5. Follow-Up

The patient experienced complete recovery of renal and liver function and was discharged home with marked improvement in volume and functional status.

## 3. Discussion

**Figure 2:**
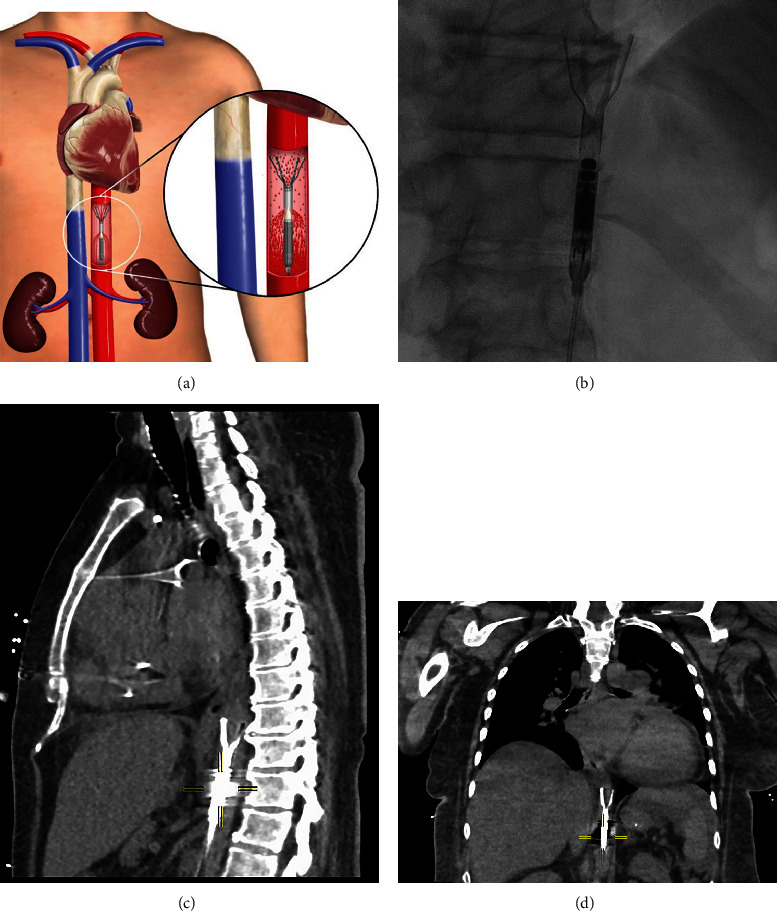
Visualization of the Aortix™ novel percutaneous mechanical circulatory support device placement in the abdominal aorta. (a) The Aortix™ device is inserted percutaneously through a common femoral artery approach and placed in the intra-abdominal aorta above the renal arteries. (b) Visualization of the device under fluoroscopy in our patient. (c) Visualization of the device (yellow marker) on a CT sagittal cross-sectional image in our patient. (d) Visualization of the device (yellow marker) on a CT coronal cross-sectional image in our patient (permission granted from Procyrion for use of copyright images). CT = computed tomography.

**Figure 3:**
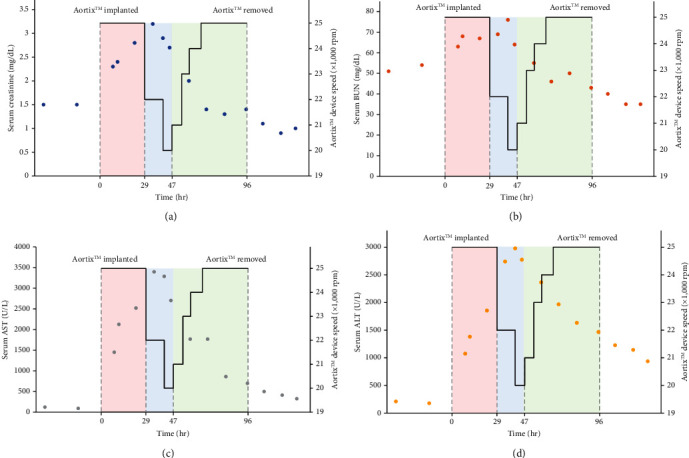
Graphic representation of end-organ perfusion as a function of the Aortix™ device speed in our patient, demonstrating titration of the device speed leads to persistent improvement in biomarker levels. (a) The relationship between serum creatinine and Aortix™ device speed over time. (b) The relationship between serum blood urea nitrogen and Aortix™ device speed over time. (c) The relationship between serum aspartate aminotransferase and Aortix™ device speed over time. (d) The relationship between serum alanine aminotransferase and Aortix™ device speed over time. Refer to Table 1 for numerical representation. AST = aspartate aminotransferase; ALT = alanine aminotransferase; BUN = blood urea nitrogen; hr = hours; rpm = rotations per minute.

Aortix™ is a novel pMCS device in clinical trials for heart failure patients on medical therapy, who have been hospitalized for acute decompensation with cardiorenal syndrome. It operates as an axial rotational blood acceleration pump, delivered via a 21 F system through a preclosed common femoral artery and anchored by atraumatic nitinol struts in the descending aorta above the renal artery and below the superior mesenteric artery [1–3] (Figure 2). Based on the mechanics of the device, it has been reported to optimally accelerate blood forward at a nominal rotational speed of 25,000 rpm. This device has demonstrated efficacy in enhancing perfusion of the kidneys to facilitate diuresis without the penalty of renal injury [2–5]. Its long-term safety and durability has been evaluated in both animal models and humans, with its first human pilot study demonstrating positive results [2–6]. Prior to this case, a single unmodulated nominal setting was prescribed. This case demonstrates several novel findings: (i) excess rotational speed causing inadvertent hypoperfusion is possible, (ii) speed modulation by titrating rpm may permit patient-specific optimization, and (iii) gradual up-titration to the nominal rotation speed setting may permit greater receptivity to the device on a patient-specific basis.  Figure 3 demonstrates the correlation of speed modulation with markers of renal and hepatic perfusion, with initial worsening, followed by titration-mediated resolution. This phenomenon has never before been described.

## 4. Conclusion(s)

This is the first documented case of end-organ hypoperfusion secondary to accelerated flow velocities caused by an appropriately positioned Aortix™ pMCS device. We also demonstrate the novel finding that the nominal rotational speed setting may not be suitable in some patients and that graded patient-specific speed modulation may optimize achievement of clinical targets. Parameters to guide speed modulation should be considered in the future study of this device.

## Figures and Tables

**Table 1:** Laboratory and hemodynamic data during the patient's hospital course as it relates to pre-Aortix™ device implantation, during implantation, and after device removal. Refer to Figure 3 for graphic representation.

Time from Aortix™ device implantation (hours)	Aortix™ device speed (×1,000 rpm)	Serum creatinine (mg/dL)	Serum BUN (mg/dL)	Serum AST (U/L)	Serum ALT (U/L)	Right atrial pressure (mmHg)	Pulmonary capillary wedge pressure (mmHg)
Pre-device implantation	N/A	1.5	54	92	178	18	33
9	25	2.3	63	1450	1074	18	23
12	25	2.4	68	2125	1381	19	23
23	25	2.8	67	2519	1854	23	29
35	25	3.2	69	3396	2740	14	21
42	22	2.9	76	3286	2978	10	19
46	20	2.7	64	2702	2773	9	16
59	24	2.0	55	1768	2364	5	16
70	25	1.4	46	1768	1966	10	22
82	25	1.3	50	861	1632	9	20
96	25	1.4	43	698	1466	7	15
Post-device removal	N/A	1.1	40	499	1227	14	24
119	N/A	0.9	35	413	1143	15	20
129	N/A	1	35	326	937		
251	N/A	1.3	43	61	256		

AST = aspartate aminotransferase; ALT = alanine aminotransferase; BUN = blood urea nitrogen; rpm = rotations per minute.

## Data Availability

All relevant patient data is provided in the manuscript.

## Abbreviations

RHC: Right heart catheterization
RAP: Right atrial pressure
PAP: Pulmonary artery pressure
PAWP: Pulmonary artery wedge pressure
CO: Cardiac output
CI: Cardiac index
CT: Computed tomography
TTE: Transthoracic echocardiogram
pMCS: Percutaneous mechanical circulatory support.
